# Aberrant Epicardial Adipose Tissue Extracellular Matrix Remodeling in Patients with Severe Ischemic Cardiomyopathy: Insight from Comparative Quantitative Proteomics

**DOI:** 10.1038/srep43787

**Published:** 2017-03-03

**Authors:** Ding-Sheng Jiang, Hao-Long Zeng, Rui Li, Bo Huo, Yun-Shu Su, Jing Fang, Qing Yang, Li-Gang Liu, Min Hu, Cai Cheng, Xue-Hai Zhu, Xin Yi, Xiang Wei

**Affiliations:** 1 Division of Cardiothoracic and Vascular Surgery, Tongji Hospital, Tongji Medical College, Huazhong University of Science and Technology, Wuhan 430030, China; 2 Key Laboratory of Organ Transplantation, Ministry of Education, Tongji Hospital, Tongji Medical College, Huazhong University of Science and Technology, Wuhan 430030, China; 3 Key Laboratory of Organ Transplantation, Ministry of Health, Tongji Hospital, Tongji Medical College, Huazhong University of Science and Technology, Wuhan 430030, China; 4 Department of Laboratory Medicine, Tongji Hospital, Tongji Medical College, Huazhong University of Science and Technology, Wuhan 430030, China; 5 College of life science, Wuhan University, Wuhan, Wuhan 430072, China; 6 Department of Cardiology, Renmin Hospital of Wuhan University, Wuhan 430060, China; 7 Cardiovascular Research Institute, Wuhan University, Wuhan 430060, China; 8 Hubei Key Laboratory of Cardiology, Wuhan 430060, P.R. China

## Abstract

There is ample evidence indicating that epicardial adipose tissue (EAT) volume and thickness is positively associated with coronary artery disease (CAD). However, the exact pathological changes in the human EAT after myocardial ischemia remains largely unclear. In the current study, we applied a comparative quantitative proteomics to elucidate the altered biological processes in the EAT of ischemic cardiomyopathy (ICM) patients. A total of 1649 proteins were successfully quantified in our study, among which 165 proteins were significantly changed (ratio <0.8 or >1.2 fold and *p* < 0.05 in both repetitions) in EAT of ICM individuals. Gene ontology (GO) enrichment analysis revealed that cardiac structure and cellular metabolism were over-represented among these regulated proteins. The hypertrophic cardiomyopathy, adrenergic signaling in cardiomyocytes, extracellular matrix (ECM)-receptor interaction, phagosome, Glycolysis/Gluconeogenesis, and PPAR signaling pathway were highlighted by the KEGG PATHWAY analysis. More importantly, we found that the proteins responsible for extracellular matrix organization were dramatically increased in EAT of ICM patients. In addition, the picrosirius red (PSR) staining results showed that the collagen fiber content was prominently increased, which indicated the EAT of ICM individuals underwent extracellular matrix remodeling and ERK1/2 activation maybe responsible for these pathological changes partially.

Epicardial adipose tissue (EAT), located between the myocardium and the visceral pericardium, is the visceral fat depot of the heart^1^. EAT mainly distributes at atrioventricular and interventricular grooves, coronary arteries and their major branches, while also expanding from the epicardial surface into the myocardium^2^. No fascial boundaries are found between EAT and myocardium, which indicates that they share a microcirculation^3^. Ample evidence demonstrate that EAT thickness and volume are closely correlated with coronary artery disease (CAD)^4^, atrial fibrillation^5^, heart failure^6^, metabolic syndrome^7^, insulin resistance^8^, and fatty liver disease^3^. In particular, EAT volume is associated with the progression of atherosclerosis and coronary artery calcification^9^. Furthermore, increased EAT volume is the independent risk factor and directly correlated with CAD^10^. However, the pathologic changes of EAT, and how EAT directly affects the pathological process of CAD or even ischemic cardiomyopathy (ICM) still remains largely unknown.

Previous research were demonstrated that EAT affects the heart in a variety of ways^3^,^11^. Under physiological conditions, EAT could release free fatty acid (FFA) to meet energy needs of the heart^12^, as well as adipokines with anti-atherogenic and anti-inflammatory effects^13^,^14^. In addition, EAT provides direct heat to the myocardium and protects the coronary artery against torsion^15^,^16^. However, under pathological conditions, EAT serves as an active secretory organ to release pro-inflammatory adipokines that promote harmful coronary artery and myocardial changes^17^. In CAD patients, infiltration of M1/M2 macrophages and pro-inflammatory cytokines were dramatically increased in EAT, which positively correlated with the severity of CAD^18^,^19^. Chechi *et al*. showed that in patients with CAD, the expression levels of brown fat like genes in EAT was correlated with circulating lipid levels^20^, which might partially provide clues for the way that EAT affects the progression of CAD. Reactive oxygen species production also remarkably increased in EAT of CAD patients^21^. Nevertheless, further basic research were needed to clarify more sophisticated functions and pathological changes of EAT in ICM patients. A comprehensive investigation of the EAT encompassing all proteins may provide a more robust data to partially answer this question.

In the present study, we applied a comparative quantitative proteomics analysis in EAT of normal or ICM patients to decipher the pathological changes at protein level. Our results indicated that cytoskeletal, metabolic, and inflammation response proteins, especially extracellular matrix (ECM) proteins were aberrant expression in EAT of ICM patients, which may regulate by ERK1/2 activation.

## Results

### Patient characteristics

Twenty-eight human samples (sixteen of ICM patients and twelve of normal controls) were included in the present study. The clinical features of the normal donors and ICM patients were summarized in Table S1. The ICM patients with 57.8 ± 1.26 years of mean age, 93.8% male, and 24.7 ± 1.16 kg/m^2^ of body mass index. There were 7 and 5 patients with hypertension or diabetes mellitus, 6 and 7 patients having drinking or cigarette history, respectively. The heart rate of these patients were 78.4 ± 6.34 bpm, and the systolic and diastolic blood pressure were 112.3 ± 5.70 mmHg and 69.3 ± 5.45 mmHg respectively. The dilated left ventricle and poor cardiac function of ICM patients were evidenced by 63 ± 3.23 mm of left ventricular end-diastolic dimension (LVEDD) and 33.4 ± 3.10% of left ventricular ejection fraction (LVEF). In addition, the concentration of blood glucose was 8.5 ± 1.06 mmol/l.

### Histology analysis of EAT in normal and ICM individuals

**Figure 1:**
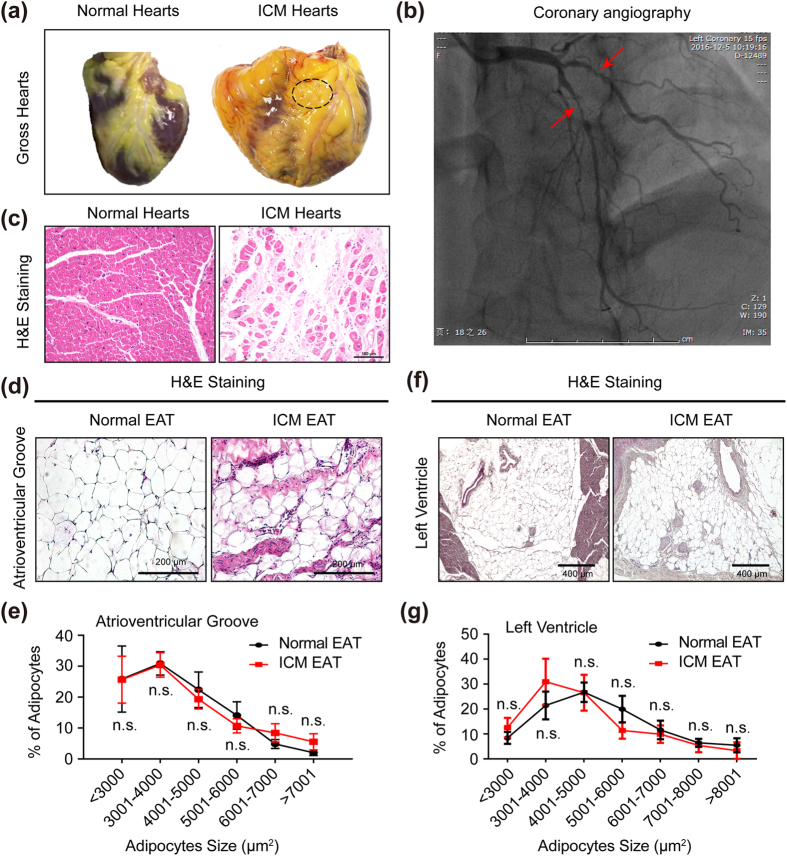
The essential features of EAT in normal donors and ICM patients. (**a**) The gross hearts of normal donor and ICM patient, and black dotted circle indicated the site of EAT we harvested (n = 12–16 samples); (**b**) The represented image of coronary angiography of ICM patients; (**c**) H&E staining of myocardium in left ventricle (n = 12–16 samples, scale bar, 100 μm); (**d**,**f**) H&E staining of EAT in atrioventricular groove (**d**) and left ventricle (**f**) of normal donors and ICM patients (n = 12–16 samples, left panel scale bar, 200 μm; right panel scale bar, 400 μm); (**e,g**) The statistical results of adipocyte size in atrioventricular groove EAT (**e**) and left ventricle EAT (**g**) of normal donors and ICM patients (n = 12–16 samples). n.s. indicated no significance.

In correlation with the literature reported, aberrant increased EAT and cardiomyocytes loss were observed in the heart of ICM patients (Fig. 1a,c). As indicated by black dotted circle, the EAT located in the atrioventricular groove was isolated in order to perform further experiments (including proteomics, western blots, real-time PCR and immunohistochemical analysis) in this study (Fig. 1a). Coronary angiography showed obvious stenosis of left anterior descending artery and diagonal branch in the presented ICM patient who has higher serum cardiac troponin I (cTnI), creatine kinase-MB (CK-MB), N-terminal-pro-B-type natriuretic peptide (NT-proBNP) concentration (Fig. 1b and Table S1). H&E staining showed that the size of adipocytes had no significant difference between normal EAT and ICM EAT, neither at atrioventricular groove (Fig. 1d,e) nor left ventricle (Fig. 1f,g).

### 165 proteins differentially expressed in the EAT of ICM patients

**Figure 2:**
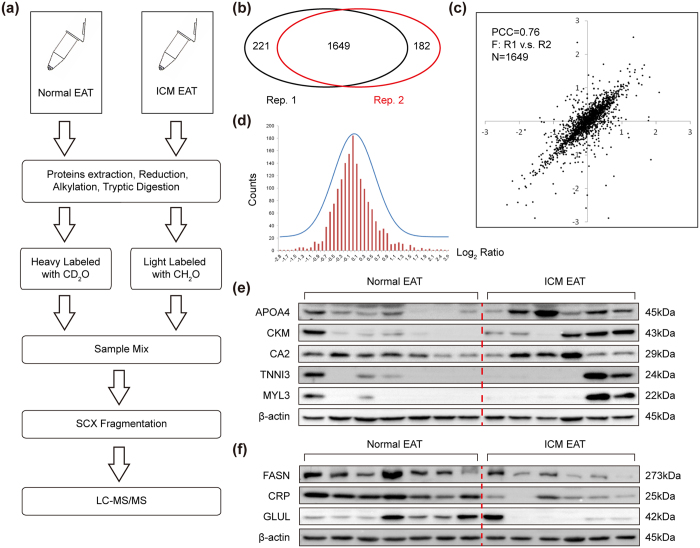
Experimental workflow and verification of quantitative proteomics. (**a**) Flowchart of the quantitative proteomics experiments; (**b**) A total of 2052 proteins was successfully quantified in the two experiment replicates, among which 1649 proteins were overlapped; (**c)** Pearson Correlation analysis shows a Pearson Correlation Coefficient (PCC) of 0.76 for the 1649 overlapping proteins in the two replicates; (**d)** Gaussian distribution of the quantitative data (Log_2_ (Ratio)) shared by the two experimental replicates, the mean value was 0.0035 and SD value was 0.538; (**e)** The protein levels of APOA4, CKM, CA2, TNNI3, and MYL3 in EAT of normal donors and ICM patients (n = 6–7 samples); (**f)** The decreased proteins (FASN, CRP, and GLUL) confirmed by western blots (n = 6–7 samples). The β-actin was served as loading control.

Since EAT volume and thickness were positive correlation with CAD^3^,^4^,^22^, it was critical to comprehensively reveal the pathological changes of EAT at protein level. Therefore, comparative quantitative proteomics analysis of EAT obtained from normal donors (heavy labeled with CD_2_O) and ICM patients (light labeled with CH_2_O) was performed by liquid chromatograph-mass spectrometer (LC-MS/MS) (Fig. 2a). Two independent repetition experiments were performed, which included seven normal EATs and eight ICM EATs, and a total of 1649 EAT proteins were quantified in both repetitions (Fig. 2b). Pearson correlation coefficient (PCC) analysis showed good repeatability between two repetitions, as evidenced by *r* = 0.76 (Fig. 2c). A Gaussian distribution of the shared quantitative data (as log_2_ (Ratio)) was further analyzed, showing a reasonable ratio distribution (Fig. 2d). All the quantified proteins were summarized in Table S2. Among the whole quantitative dataset, seventy-two proteins (average ratio <0.8 fold and *p* < 0.05 in both repetitions) were found to be significantly down-regulated in ICM EATs, whereas ninety-three proteins (average ratio >1.2 fold and *p* < 0.05 in both repetitions) were up-regulated (Table S3). As a way to cross-check the reliability of quantitative proteomics, several proteins expression levels were assessed using conventional western blot on the same specimens. In accordance with the results of proteomics analysis, western blot demonstrated that the protein levels of apolipoprotein A-IV (APOA4), creatine kinase M-type (CKM), carbonic anhydrase 2 (CA2), troponin I, cardiac muscle (TNNI3), and myosin light chain 3 (MYL3) were ectopic expression in ICM EATs compared with normal counterparts (Fig. 2e), while fatty acid synthase (FASN), c-reactive protein (CRP), and glutamine synthetase (GLUL) were obviously down-regulated (Fig. 2f). In this light, we were prudent to conclude that the data of comparative quantitative proteomics analysis was very reliable.

### Multiple biological processes were affected in EAT of ICM patients

**Figure 3:**
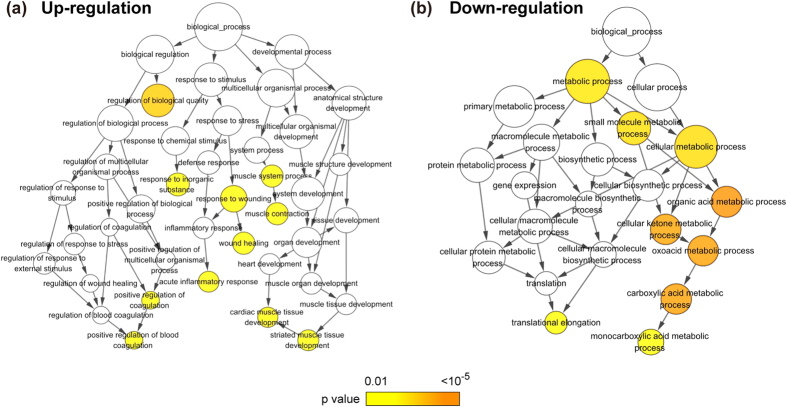
Gene ontology enrichment of the biological processes in clusters of regulated proteins. **(a**) Eleven terms including blood coagulation, inflammation and muscle contraction were over represented (p < 0.00001) in the biological process category of up-regulated proteins. (**b**) Nine terms including translation elongation and various metabolic processes were over represented (p < 0.00001) in the biological process category of down-regulated proteins. Terms are depicted as nodes connected by arrows that represent hierarchies and relationships between terms. Node size is proportional to the number of proteins assigned to a given ontology term, whereas node color represents the corrected p-value (Benjamin Hochberg false discovery rate correction) corresponding to enrichment of the term.

To ascertain the functional representation of these differentially expressed proteins, the data were further analyzed based on the gene ontology (GO) term enrichment. The results showed that the proteins up-regulated in the EAT of ICM patients were found significantly enriched among the inflammation and cardiac structure related the GO terms including regulation of biological quality, response to inorganic substance, muscle contraction, wound healing, acute inflammatory response, positive regulation of blood coagulation, and striated muscle tissue development (Fig. 3a), while down-regulated proteins were mainly enriched in metabolic process, such as organic acid metabolic process, cellular ketone metabolic process, oxoacid metabolic process, carboxylic acid metabolic process, monocarboxylic acid metabolic process, as well as translational elongation (Fig. 3b).

**Figure 4:**
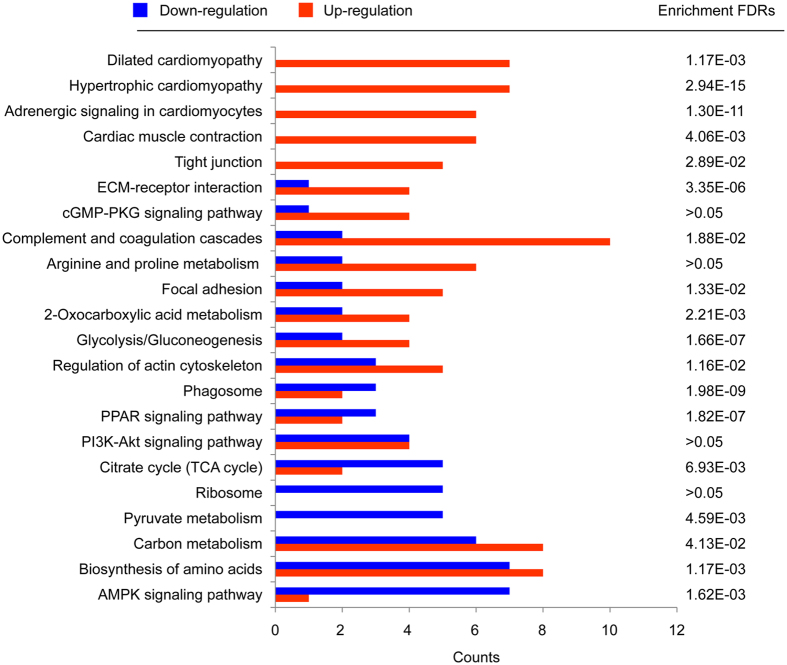
KEGG pathway analysis of regulated proteins in EATs of ICM patients. The regulated proteins were submitted to the KEGG database, respectively, for intracellular pathway analysis. A total of 22 KEGG pathways were involved, in which cardiac disease related pathways and metabolic process were mainly enriched among the down-regulated and up-regulated proteins, respectively. The pathway enrichment FDRs (p-value) of the total regulated proteins in KEGG database were also given.

To understand the intracellular pathways involvement in these differentially expressed proteins, the KEGG PATHWAY database mapping analysis was performed. Our results revealed that a total of 22 pathways were found to be involved (Fig. 4), and among them proteins involved in the cardiac muscle function related pathways like dilated cardiomyopathy (*p = *1.17E-3), the hypertrophic cardiomyopathy (*p = *2.94E-15), adrenergic signaling in cardiomyocytes (*p = *1.30E-11), and cardiac muscle contraction(*p = *4.06E-3) were mainly increased, while those involved in tricarboxylic acid (TCA) cycle (*p = *6.93E-03), Ribosome (*p > 0.05*), pyruvate metabolism (*p = *4.59E-03) and AMPK signaling pathways (*p = *1.62E-03) were mainly decreased. The p-value here represents the protein enrichment false discovery rates (FDRs) of the pathway terms (Fig. 4).

### The proteins related to ECM were ectopic expression in EAT of ICM patients

**Figure 5:**
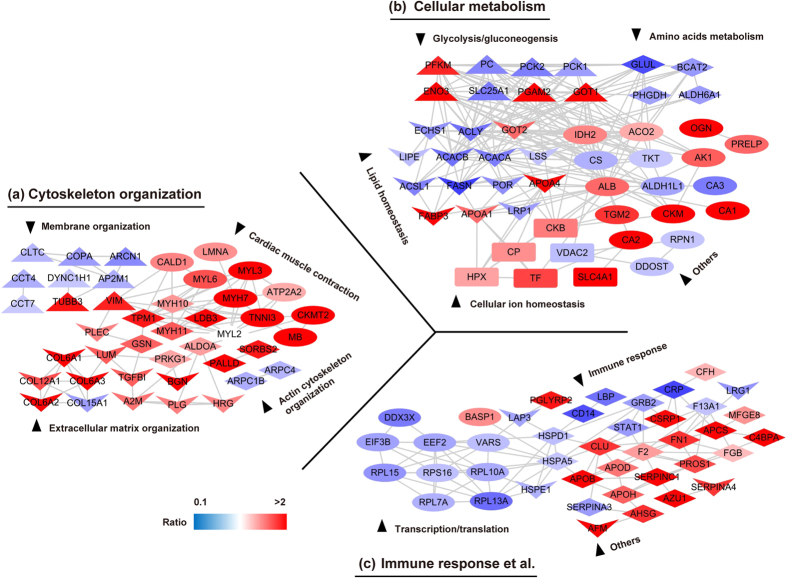
The associated networks of regulated proteins in EATs of ICM patients. The total 165 differentially expressed proteins were submitted to STRING 9.0 for network analysis. (**a–c)** Three functional subnetworks including cytoskeleton organization (**a**), cellular metabolism (**b**) and immune response (**c**), and a total of 12 functional clusters were established according to the Uniprot GeneOntoloy annotations. The node color represents the quantitative ratio.

**Figure 6:**
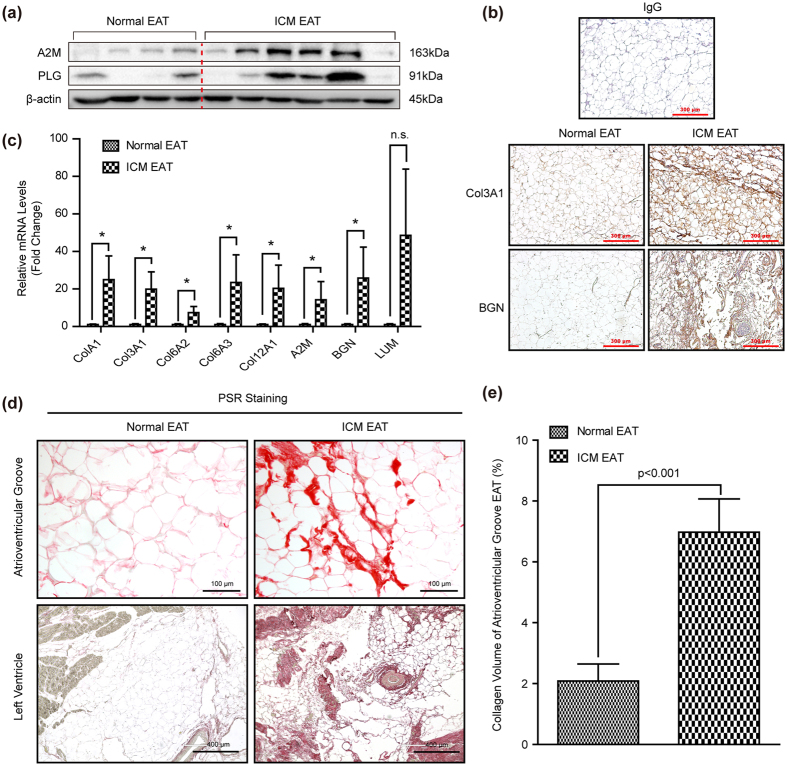
The ECM and its related proteins were significantly increased in the ICM EAT. (**a)** The protein levels of A2M and PLG was evaluated by western blots (n = 6–8 samples). The β-actin was served as loading control; (**b**) The Col3A1 and BGN protein levels and cellular location was detected by immunohistochemical staining, IgG serve as a negative control. The brown represented positive staining, and blue staining indicated nucleus (n = 6–8 samples, scale bar, 300 μm); (**c**) The mRNA levels of Col1A1, Col3A1, Col6A2, Col6A3, Col12A1, A2M, BGN, LUM were detected by real-time PCR (n = 4–9 samples); (**d)** The PSR staining in the atrioventricular groove EAT and left ventricle EAT of normal donors and ICM patients, and red staining represented ECM (n = 6–8 samples, top panel scale bar, 100 μm; bottom panel scale bar, 400 μm); (**e)** The collagen volume quantitative result of atrioventricular groove EAT (n = 12–16 samples). *p < 0.05 v.s. normal EAT, n.s. indicated no significance.

To further investigate the possible regulated signaling network among altered EAT proteome of ICM patients systematically, we established the protein interaction network based on all those differentially expressed proteins using the STRING database and the Uniprot functional annotation. Our data indicated that protein interaction network related to the three categories: cytoskeleton organization, cellular metabolism, and immune response were significantly altered in the EAT of ICM individuals (Fig. 5a–c). Most of the altered proteins involved in lipid homeostasis, amino acids metabolism, glycolysis/gluconeogenesis, as well as transcription were prominently reduced, but the majority of changed proteins participated in cellular ion homeostasis and immune response were obviously increased in ICM EATs (Fig. 5b,c). As for cytoskeleton organization, most of changed proteins associated with membrane organization were down-regulated (*e.g.* CLTC, ARCN1, COPA, and AP2M1) in EAT of ICM patients, while opposite situation was observed in the relevant proteins of cardiac muscle contraction, actin cytoskeleton organization, and ECM organization (Fig. 5a). All the proteins involved in the network analysis were summarized in Table S4. Consistent with the results of proteomics, the protein or mRNA levels of A2M, PLG, Col3A1, BGN, Col1A1, Col6A2, Col6A3, Col12A1 and LUM were significantly increased in the atrioventricular groove EAT of ICM individuals (Fig. 6a–c). More importantly, concomitant changes with the ECM related proteins, abundant ECM was detected by picrosirius red (PSR) staining in both atrioventricular groove EAT and left ventricle EAT of ICM patients (Fig. 6d,e). Therefore, all the aforementioned data indicated that severe ECM remodeling was taken place in the EAT of ICM patients.

### The ERK1/2 activation may be partially responsible for the ECM remodeling of EAT in ICM patients

**Figure 7:**
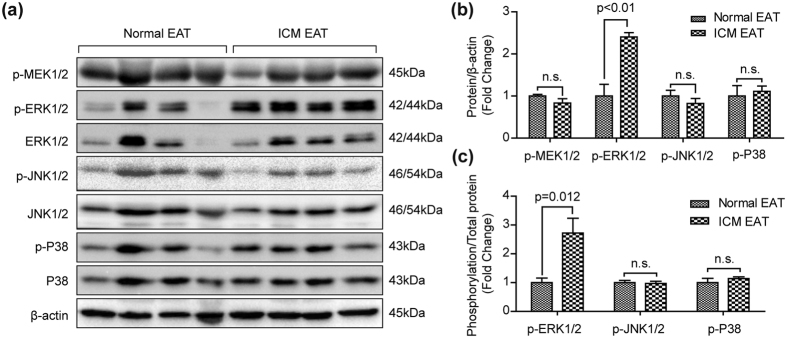
Total and phosphorylation protein levels of MAPK signaling pathway in the EAT. (**a)** Total and phosphorylation levels of MEK1/2, ERK1/2, JNK1/2, and P38 were evaluated by western blots (n = 7–8 samples). The β-actin was served as loading control. (**b**) The gray value ratio of target phosphorylation protein to β-actin; (**c**) The gray value ratio of target phosphorylation protein to its total protein. n.s. indicated no significance.

To investigate the mechanism promoting ECM accumulation in the EAT of ICM patients, the mitogen-activated protein kinase (MAPK) signaling pathway, which is closely related to cardiac fibrosis^23^, was detected in the EAT samples. There are three classic MAPK subfamilies, including ERK1/2, JNK1/2 and P38^24^. Our results demonstrated that the phosphorylation levels of MEK1/2, JNK1/2 and P38 showed no significant difference between normal and ICM EAT groups (Fig. 7a–c). However, the phosphorylated ERK1/2 was dramatically increased in the ICM EATs compared with normal EATs (Fig. 7a–c). These results indicated that ERK1/2, but not JNK1/2 and P38 activation may be involved in ECM remodeling in the EAT of ICM patients.

## Discussion

Since EAT volume and thickness were closely related to CAD^9^,^10^, it’s important to ascertain the changed biological processes of EAT in CAD or ICM patients. In the present study, we identified 165 differentially expressed proteins in the EAT of ICM patients, which were associated with cytoskeleton organization, cellular metabolism, and immune response respectively. Notably, compared with normal EAT, the ECM proteins were aberrant expression in EAT of ICM, which may partially regulate by ERK1/2 activation.

EAT, a kind of adipose tissue, has the general characteristics of adipose tissue, including energy storage and metabolism, endocrine/paracrine cytokines, thermal regulation, and protecting organs from mechanical damage^25^. Though we did not yet know the exact mechanisms on how increased EAT affects the pathological changes of CAD or even ICM, considerable advances have been made in recent years. Uchida *et al*. demonstrated that pericoronary adipose tissue as storage and possible supply site for oxidized low-density lipoprotein (oxLDL) and high-density lipoprotein (HDL) in coronary arteries, and CD68(+)-macrophages or vasa vasorum were responsible for oxLDL reaching to coronary intima, while HDL was supplied to coronary intima only by vasa vasorum^26^. Excessive oxLDL would accelerate formation of atheromatous plaque and CAD development. Compared with non-CAD patients, the polarization of M1/M2 macrophages was shifted toward a pro-inflammatory state in the EAT of CAD patients^18^. Multiple pro-inflammatory and atherogenic cytokines (*e.g.* MCP-1, IL-1β, IL-6, TNFα, PAI-1) were secreted by EAT in excess into the adjacent myocardium and coronary arteries^3^. Pericardial fluid, containing many kinds of peptide hormones and growth factors, is directly contact with the EAT and the myocardium^27^,^28^. Angiotensin-converting enzyme (ACE), bradykinin (BK) were detected in the pericardial fluid of coronary artery disease (CAD) patients^29^, which played a function on adjacent coronary artery or myocardium. However, the source of ACE and BK was unclear, and EAT may contribute to their accumulation in the pericardial fluid. Higher levels of reactive oxygen species were observed in the EAT of CAD patients^21^. In severe CAD subjects, the transcriptional level of gene sets related to intracellular trafficking, proliferation/transcription regulation, protein catabolism, innate immunity/lectin pathway, and ER stress were downregulated^30^. In addition, the research of EAT on atrial cardiomyopathy and atrial fibrillation had made some progress recently. Venteclef *et al*. demonstrated that the secretome from EAT promotes fibrosis of the atrial myocardium through the secretion of adipo-fibrokines such as Activin A^31^. In our current study, our results indicated that, a hitherto unrecognized finding, the ECM was significantly increased in the EAT of ICM patients and ERK1/2 activation (Figs 5, 6, 7) may participate in this biology process. These findings indicated that EAT affected atria and ventricles in a similar fashion, at least partially.

Previous studies indicated that ECM, mainly composed of structural proteins (*e.g.* collagens, osteopontin), adhesion proteins, and proteoglycans, was crucial for the structural integrity of adipocytes and its function^32^. Henegar *et al*. demonstrated that a higher amount of fibrosis was observed in the obese subcutaneous white adipose tissue (WAT) than that of lean human beings^33^. Similarly, increased collagens were detected in the epididymal fat pads of *db/db* mice (a well-known genetic model of obesity)^34^. The changes, such as components or content, happened in the ECM could affect intracellular signaling pathways of adipose and adjacent cells through its receptors (*e.g.* integrins and CD44), and finally trigger the necrosis of adipocytes, physical restriction on adipose tissue expansion, and adipose inflammation in WAT^32^,^35^. These research achievements on WAT may provide clues for EAT study. Ample evidence demonstrated that CAD severity was positively associated with EAT volume or thickness^4^,^10^,^22^,^36^. However, it remains unclear whether the ECM in EAT of ICM patients altered. Our present study demonstrated that compared with normal EAT, the ECM proteins, such as Col3A1, Col1A1, Col6A1, Col6A2, Col6A3, Col12A1, TGFBI, LUM, A2M, BGN, were remarkably increased in the EAT of ICM patients (Figs 5a and 6a–c). Furthermore, a large number of collagen fibers deposition was observed in the EAT of ICM patients via PSR staining (Fig. 6d,e). However, in pathologic conditions, more research is nevertheless necessary to clarify the contribution of ECM modifications and the consequence of fibrous depots on EAT and cardiac function.

Since fibrosis not only impaired adipocyte functionality, but also limited adipocyte hypertrophy^37^, we assessed the adipocyte size in EAT. As was recognized that the EAT volume and thickness were obviously increased in the CAD and ICM patients^4^,^10^, this conclusion was verified in our study (Fig. 1a). Generally, the increased fat volume, thickness or weight was resulted from increased adipocyte size (hypertrophy) and/or number (hyperplasia)^38^. Adipocyte hypertrophy, leading to WAT dysfunction (*e.g.* abnormal lipid storage and adipogenesis, impaired secretion of adipokines, exacerbated fibrosis deposition and insulin resistance), was a hallmark of WAT enlargement in obesity^38^. However, in the present study, our data showed that the adipocyte size in EAT had no significant difference between normal donors and ICM patients (Fig. 1e,g). These data indicated that the increased EAT volume and thickness in ICM individuals may not due to adipocyte hypertrophy. Previously published literature were demonstrated that fibrosis might affect the expandability of WAT by physically limiting adipocyte hypertrophy^39^. The adipocytes in WAT exhibited hypertrophy in *ob/ob* mice lacking collagen VI which were absence of fibrotic deposits^34^. In addition, the adipocyte size was profoundly affected by genetic ablation of collagens or remodelling enzymes^38^. Given that, the increased EAT volume or thickness of ICM patients maybe the result of hyperplasia. However, further research is needed to elucidate whether hyperplasia is the reason for increased EAT volume or thickness in ICM patients in the future.

Most of currently published studies were using subcutaneous or omental adipose tissue as control group, but not normal EAT^18^,^20^,^21^. As we known, compared with EAT, subcutaneous or omental adipose tissue have a different gene profile and function in normal conditions. These differentially expressed genes may not contribute to the pathological processes of diseases. Therefore, in our present study, the ICM and normal EAT were applied to comparative quantitative proteomics to elucidate the events occurred in the EAT of ICM patients at protein level. However, because of the limitation of sensitivity of mass spectrometry, lots of low abundance proteins may have been missed in our study. The post-translational modification of proteins, such as activation by phosphorylation, was not studied in the present study. In addition, a relatively small number of samples were included in the present study. Therefore, more research and biological methods were needed to make great progress in the field of EAT.

## Methods and Materials

### Patients and tissue samples

Approval was got from Tongji Hospital, Tongji Medical College, Huazhong University of Science and Technology Review Board in Wuhan, China. All the procedures involving human samples conformed to the principles outlined in the Declaration of Helsinki. Participation was voluntary and informed consent was obtained in all cases. EAT samples of ICM were collected from patients undergoing heart transplantation due to irreversible heart failure caused by myocardial infarction. Normal EAT samples were obtained from the normal heart donors who died in accidents but whose hearts were not suitable for transplantation for noncardiac reasons^40^,^41^,^42^. Samples were flash frozen and processed for proteomic analysis, western blot and real-time PCR.

### Extraction and digestion of epicardial adipose proteins

EAT biopsies from eight patients with ICM and seven healthy controls were used for proteomics analysis. Tissues were washed in PBS immediately after removal and cut into small pieces, then directly frozen in liquid nitrogen until needed. Homogenization of EAT were processed (a pooled approach to minimize the biological variations) using the HNTG lysis buffer (50 mM HEPES, 150 mM NaCl, 10% glycerol, 1% Triton X-100) with a glass-Teflon homogenizer. The resulted samples were then vortex in cold room for 30 min and centrifuge at 12000 g, 4 °C for 20 min. the fat would form a layer at the top and the cell debris would form a pellet. Avoiding the cell debris layer and pipetting going under the fat to pull off the clear/reddish brown lysate, repeat this last step twice. The epicardial adipose proteins were collected and divided into aliquots for two independent experimental replicates to be conducted.

The in-solution digestion of EAT proteins was performed as described in a previous study^43^. Proteins were precipitated by mixing with 50% acetone/50% methanol/0.1% acetic acid, followed by centrifugation at 2000 g for 20 min. The protein pellets were re-suspended with 8 M Urea/4 mM CaCl_2_/0.2 M Tris-HCl, pH 8.0, and reduced with 10 mM DTT at 50 °C for 30 min and then alkylated with 40 mM iodoacetamide in the dark for 30 min. After measuring protein concentrations via Bradford assay, the proteins were digested with trypsin at a ratio of 1:50 (trypsin/protein w/w). The digested peptides were desalted using a SepPak C18 cartridge (Waters) and dried with a SpeedVac.

### Stable isotope dimethyl labeling and strong cation exchange (SCX) fractionation

Desalted peptides were re-suspended in 0.1 M sodium acetate, pH 6.0. Next, 4% formaldehyde (CH_2_O, which serves as “light labeled”) was added to the peptides from ICM EAT, and 4% deuterated formaldehyde (CD_2_O, which serves as “heavy labeled”) was added to the peptides extracted from normal EAT. After mixing, 0.6 M sodium cyanoborohydride (NaBH_3_CN) was added, and the mixtures were incubated at room temperature (20 ± 2 °C) for 1 hour. The samples were then quenched by adding 1% ammonium hydroxide, followed by the addition of 5% formic acid. After labeling, the peptides were mixed at a ratio of 1:1 and desalted again prior to separation via SCX chromatography.

For SCX fractionation, Buffer A contained 5 mM KH_2_PO_4_, pH 2.7, in 20% acetonitrile/80% ddH_2_O. Buffer B contained 5 mM KH_2_PO_4_ and 0.5 M KCl, pH 2.7, in 20% acetonitrile/80% ddH_2_O. SCX was performed on a polysulfoethyl column (2.1 × 50 mm, 5 μm × 200 Å) using a KCl gradient from 0 to 0.5 M at a flow rate of 0.2 ml/min. During a 60 min gradient elution, nearly twelve fractions were collected and desalted with a C18 ZipTip (Millipore) prior to MS analysis.

### LC-MS/MS and data processing

All ESI-based LC-MS/MS experiments were performed on a TripleTOF 5600+ System coupled with an Ultra 1D Plus nano-liquid chromatography device (SCIEX, USA). Dried peptides were dissolved in 0.1% formic acid/2% acetonitrile/98% H_2_O, loaded onto a C18 trap column (5 μm, 5 × 0.3 mm, Agilent Technologies) at a flow rate of 5 μL/min, and subsequently eluted from the trap column over the C18 analytic column (75 μm × 150 mm, 3 μm particle size, 100 Å pore size, Eksigent) at a flow rate of 300 nL/min in a 100 min gradient. The mobile phase consisted of two components: component A was 3% DMSO/97% H_2_O with 0.1% formic acid, and component B was 3% DMSO/97% acetonitrile with 0.1% formic acid. The information dependent acquisition (IDA) mode was used to acquire MS/MS data. Survey scans were acquired in 250 ms and 40 product ion scans were collected at 50 ms/per scan. The precursor ion range was set from m/z 350 to m/z 1500, and the product ion range was set from m/z 100 to m/z 1500.

The generated raw MS spectra was analyzed with Proteinpilot 4.5 software (SCIEX, USA) by using the Paragon algorithm. The Uniprot_Human_201510 database (downloaded from UniProt) was used. The data analysis parameters were as follows: Sample type: Dimethyl +0, +4, +8 (Peptide Labeled); Cys Alkylation: Iodoacetamide; Digestion: Trypsin; Instrument: TripleTOF 5600; Special Factors: Urea denaturation; ID Focus: Biological modifications, Amino acid substitution; Search Effort: Thorough ID; Detected Protein Threshold [Unused ProtScore (Conf)]: 1.3 (95.0%). The false discovery rates (FDRs) of the peptide-spectra matches determined by a decoy database search were set to 1.0%. Proteins were considered to be successfully identified when at least two correct assigned peptide (95% confidence) was obtained.

Two independent experimental replicates were generated and analyzed. The Pearson’s correlation coefficiency for protein ratios between two replicates was measured. The protein ratio values used in the bioinformatics analysis described below were the means of the two experimental replicates.

### Bioinformatics

Biological Networks Gene Ontology (BiNGO) 3.03 was used to calculate the gene ontology (GO) term enrichment of significantly up- or down-regulated proteins (defined as quantitative ratio >1.2 or <0.8 and p-value < 0.05 in both replicates) and determine significantly under- and over-represented functional GO categories. The Cytoscape network visualization platform (http://www.cytoscape.org/) implementing the latest release of the BiNGO plug-in was used to identify proteins that were annotated on the basis of biological process categories. The analysis was conducted using the default BiNGO Homo sapiens database. Statistical significance was determined by means of hypergeometric analysis, followed by Benjamini and Hochberg’s false discovery rate correction (p < 0.00001)^44^.

The intracellular pathway analysis was performed by using the KEGG PATHWAY database via the KEGG automatic annotation server (http://www.genome.jp/kegg/)^45^. The differentially expressed proteins matched in the KEGG PATHWAY database were counted and processed by Microsoft Office Excel.

For protein interaction network analysis of regulated proteins involved in various pathways, the Uniprot functional annotations (http://www.uniprot.org/uniprot) were used to classify the proteins into several clusters. Based on the quantified MS results, proteins matched in any clusters were extracted and submitted to STRING 9.0 (the Search Tool for the Retrieval of Interacting Genes/Proteins) to qualify the physical and functional interactions of these proteins. The proteins and their interactions were then uploaded to Cytoscape (version 2.8.3) for data visualization.

### Western blot analysis

Western blot was performed as previously reported^40^,^46^,^47^,^48^. The Phospho-MEK1/2 (Ser217/221) (#9154), ERK1/2 (#4695), Phospho-p44/42 MAPK (Erk1/2) (Thr202/Tyr204) (#4370), JNK/SAPK (#9258), Phospho-SAPK/JNK (Thr183/Tyr185) (#9255), p38 MAPK (#8690), Phospho-p38 MAPK (Thr180/Tyr182) (#4511), and β-actin (#8457) antibodies were purchased from Cell Signaling Technology. Antibodies against APOA4 (D221656), CKM (D260075), CA2 (D120344), TNNI3 (D120341), MYL3 (D122718), CRP (D120482), FASN (D262701), GLUL (D122427), A2M (D162821), and PLG (D262067) were obtained from BBI Life Sciences. All the primary antibodies were diluted as 1:1000. The protein signals were detected by using a ChemiDoc XRS+ Imaging System (Bio-Rad), and then the gray value of proteins were analyzed by Image lab software (version 5.2.1, Bio-Rad).

### Real-Time PCR

The real-time PCR (RT-PCR) was performed as our previously reported^40^. The primers used in the present study were listed as follows. Col1A1-forward (5′-GAGGGCCAAGACGAAGACATC-3′), Col1A1-reverse (5′-CAGATCACGTCATCGCACAAC-3′); Col3A1-forward (5′-TTGAAGGAGGATGTTCCCATCT-3′), Col3A1-reverse (5′-ACAGACACATATTTGGCATGGTT-3′); Col6A2-forward (5′-GTCATCTCGCCGGACACTAC-3′), Col6A2-reverse (5′-GGTGTCCAGCACGAAGTACA-3′); Col6A3-forward (5′-CCTGTGTGCATTCATCCGTG-3′), Col6A3-reverse (5′-ACATGGTTCTGGGCTCATCG-3′); Col12A1-forward (5′-TTCCTCGTGGATGGCTCTTG-3′), Col12A1-reverse (5′-ATCAATGGCATCCCCTG TCA-3′); A2M-forward (5′-TTGAAGAGCCTCACACGGAG-3′), A2M-reverse (5′-TCCAGCATCTTCAGACAGGC-3′); BGN-forward (5′-CTGTCACACCCACCTACAGC-3′), BGN-reverse (5′-TGAAGTCATCCTTGCGGAGC-3′); LUM-forward (5′-CAACGAACTGGCTGATAGTGG-3′), LUM-reverse (5′-CTTGG AGTAGGATAATGGCCCC-3′); GAPDH-forward (5′-GAGTCAACGGATTTGGTCGT-3′), GAPDH-reverse (5′-TTGATTTTGGAGGGATCTCG-3′).

### Histology staining

The surgically resected EAT specimens were fixed using 4% neutral buffered formalin. After 48 hours, the EAT were dehydrated, then embedded in paraffin using standard histological procedures. Subsequently, the EAT were sectioned at 3μm, and the sections were stained with hematoxylin-eosin (H&E) and picrosirius red (PSR) as our previously reported^23^,^49^. PSR was used to evaluate ECM collagen deposition.

### Immunohistochemical analysis

The sections of atrioventricular groove EAT were stained with BGN (D121985) and Col3A1 (D220454) which were obtained from BBI Life Sciences to evaluate their protein levels and cellular localization by using minor revised standard immunohistochemical analysis procedures as we previously reported^40^.

### Statistical analysis

The data were represented as mean ± standard error (SEM). Student’s two-tailed t-test was used to compare the means of two groups. *p* < 0.05 is considered as statistical significance. The SPSS software (version 13.0) was used to perform all the statistical analysis of present study.

## Additional Information

**How to cite this article**: Jiang, D.-S. *et al*. Aberrant Epicardial Adipose Tissue Extracellular Matrix Remodeling in Patients with Severe Ischemic Cardiomyopathy: Insight from Comparative Quantitative Proteomics. *Sci. Rep.* **7**, 43787; doi: 10.1038/srep43787 (2017).

**Publisher's note:** Springer Nature remains neutral with regard to jurisdictional claims in published maps and institutional affiliations.

## Supplementary Material

Supplementary Dataset 1

Supplementary Dataset 2

Supplementary Dataset 3

Supplementary Dataset 4
