# Supplementary material for: Aberrant Epicardial Adipose Tissue Extracellular Matrix Remodeling in Patients with Severe Ischemic Cardiomyopathy: Insight from Comparative Quantitative Proteomics
Source: Sci Rep. 2017 Mar 3;7:43787. doi: 10.1038/srep43787 (PMC5335613; doi:10.1038/srep43787)
Supplement: Supplementary Dataset 1 [file srep43787-s1.doc]

**Aberrant Epicardial Adipose Tissue Extracellular Matrix Remodeling in Patients with Severe Ischemic Cardiomyopathy: Insight from Comparative Quantitative Proteomics**

Ding-Sheng Jiang^1,2,3#^, Hao-Long Zeng^4#^, Rui Li^1^, Bo Huo^1^, Yun-Shu Su^1^, Jing Fang^1^, Qing Yang^5^, Li-Gang Liu^1^, Min Hu^1^, Cai Cheng^1^, Xue-Hai Zhu^1,2,3^, Xin Yi^6,7,8^*, Xiang Wei^1,2,3^*

**Supplementary table S1. The information of normal donors and patients with severe ischemic cardiomyopathy (ICM)**.

| **Characteristics** | **Normal donors** | **ICM patients** |
| --- | --- | --- |
| No. | 12 | 16 |
| Age, y | 28.6±1.79 | 57.8±1.26 |
| Sex, male/total (%) | 9/12 (75%) | 15/16 (93.8%) |
| BMI (kg/m^2^) | 24.0±0.57 | 24.7±1.16 |
| Medical history |  |  |
| Hypertension | 0 (0%) | 7 (43.75%) |
| Diabetes mellitus | 0 (0%) | 5 (31.25%) |
| Drinking history | 1 (8.33%) | 6 (37.5%) |
| Cigarette smoker | 2 (16.67%) | 7 (43.75%) |
| LVEDD (mm) | 42.2±0.51 | 63±3.23 |
| LVEF (%) | 61.7±0.47 | 33.4±3.10 |
| Heart rate, bpm | 74.3±2.26 | 78.4±6.34 |
| Blood pressure, mmHg |  |  |
| Systolic | N/A | 112.3±5.70 |
| Diastolic | N/A | 69.3±5.45 |
| Blood glucose, mmol/l | N/A | 8.5±1.06 |
| cTnI, ng/ml | N/A | 4551.8±4544.8 |
| CK-MB, ng/ml | N/A | 39.7±20.31 |
| NT-proBNP, pg/ml | N/A | 4314.8±859.9 |

BMI: body mass index; LVEDD: left ventricular end-diastolic dimension; LVEF: left ventricular ejection fraction; cTnI: cardiac troponin I; CK-MB: creatine kinase-MB; NT-proBNP: N-terminal-pro-B-type natriuretic peptide
